# Acquired resistance to tyrosine kinase inhibitors may be linked with the decreased sensitivity to X-ray irradiation

**DOI:** 10.18632/oncotarget.23700

**Published:** 2017-12-27

**Authors:** Maxim Sorokin, Roman Kholodenko, Anna Grekhova, Maria Suntsova, Margarita Pustovalova, Natalia Vorobyeva, Irina Kholodenko, Galina Malakhova, Andrew Garazha, Artem Nedoluzhko, Raif Vasilov, Elena Poddubskaya, Olga Kovalchuk, Leila Adamyan, Vladimir Prassolov, Daria Allina, Denis Kuzmin, Kirill Ignatev, Andreyan Osipov, Anton Buzdin

**Affiliations:** ^1^ D. Rogachev Federal Research Center of Pediatric Hematology, Oncology and Immunology, Moscow 117198, Russia; ^2^ National Research Centre “Kurchatov Institute”, Centre for Convergence of Nano-, Bio-, Information and Cognitive Sciences and Technologies, Moscow 123182, Russia; ^3^ Shemyakin-Ovchinnikov Institute of Bioorganic Chemistry, Moscow 117997, Russia; ^4^ State Research Center-Burnasyan Federal Medical Biophysical Center of Federal Medical Biological Agency, Moscow 123098, Russia; ^5^ Engelhardt Institute of Molecular Biology, Russian Academy of Sciences, Moscow 119991, Russia; ^6^ Orekhovich Institute of Biomedical Chemistry, Moscow 119121, Russia; ^7^ OmicsWay Corp., Walnut, CA 91789, USA; ^8^ Clinical Center Vitamed, Moscow 121309, Russia; ^9^ Department of Biological Sciences, University of Lethbridge, Lethbridge, AB T1K3M4, Canada; ^10^ Department of Reproductive Medicine and Surgery, Moscow State University of Medicine and Dentistry, Moscow 127206, Russia; ^11^ Pathology Department, Morozov Children's City Hospital, Moscow 119049, Russia; ^12^ LLC “Solixant”, Moscow 119991, Russia; ^13^ Republic Oncological Hospital, Petrozavodsk 185000, Russia

**Keywords:** tyrosine kinase, serine and threonine kinase, X-ray irradiation, drug resistance, DNA repair

## Abstract

Acquired resistance to chemotherapy and radiation therapy is one of the major obstacles decreasing efficiency of treatment of the oncologic diseases. In this study, on the two cell lines (ovarian carcinoma SKOV-3 and neuroblastoma NGP-127), we modeled acquired resistance to five target anticancer drugs. The cells were grown on gradually increasing concentrations of the clinically relevant tyrosine kinase inhibitors (TKIs) Sorafenib, Pazopanib and Sunitinib, and rapalogs Everolimus and Temsirolimus, for 20 weeks. After 20 weeks of culturing, the half-inhibitory concentrations (IC_50_) increased by 25 – 186% for the particular combinations of the drugs and cell types. We next subjected cells to 10 Gy irradiation, a dose frequently used in clinical radiation therapy. For the SKOV-3, but not NGP-127 cells, for the TKIs Sorafenib, Pazopanib and Sunitinib, we noticed statistically significant increase in capacity to repair radiation-induced DNA double strand breaks compared to naïve control cells not previously treated with TKIs. These peculiarities were linked with the increased activation of ATM DNA repair pathway in the TKI-treated SKOV-3, but not NGP-127 cells. Our results provide a new cell culture model for studying anti-cancer therapy efficiency and evidence that there may be a tissue-specific radioresistance emerging as a side effect of treatment with TKIs.

## INTRODUCTION

Target anticancer therapy uses a new generation of drugs that selectively bind and inhibit certain protein molecules, thus interfering with tumor growth and survival. Tyrosine kinase inhibitors (TKIs) is a family of target drugs that repress oncogenic tyrosine kinase proteins such as receptors of PDGF, EGF, FGF and VEGF, RAF proteins, cKIT, KDR, RET, FLT4, ALK and the others [1]. Some TKIs repress single proteins, such as the drugs Dabrafenib and Vemurafenib that block the *V600E* mutant form of a BRAF kinase [2, 3], whereas the others may inhibit multiple molecular targets [4]. For example, the drug Sorafenib blocks the VEGFR, PDGFR and Raf family kinases, with strong preference to C-Raf than B-Raf [5].

In this study, we focused on five market leading target anticancer drugs, including three TKIs and two rapalogs (Everolimus and Temsirolimus) that inhibit serine/threonine kinase complex MTOR. They are routinely in use for ten cancer types, such as the breast cancer, lymphomas, leukemia, sarcomas, renal cancer, hepatocellular carcinoma, gastrointestinal stromal tumor, pancreatic cancer, thyroid cancer and neuroendocrine tumors (Table 1). In contrast, they were currently not officially approved by the FDA for using in ovarian cancer and in neuroblastoma [6, 7]. However, there are many clinical trials ongoing with these drugs, for example, Sorafenib and Pazopanib are now at the 2^nd^ phase trial for the ovarian cancer [8, 9].

**Table 1:** Molecular and clinical specificities of the target anticancer drugs used in this study

Drug	Molecular targets	Approved indications in oncology
Sorafenib	*BRAF, RAF1, KDR, FLT4, FLT3, PDGFRB, KIT, RET, FLT1, FGFR1*	Renal cancer, Hepatocellular carcinoma, Thyroid cancer
Pazopanib	*FLT1, KDR, FLT4, PDGFRA, PDGFRB, KIT*	Renal cancer, Sarcomas
Sunitinib	*PDGFRB, FLT1, KIT, KDR, FLT4, FLT3, CSF1R, PDGFRA*	Renal cancer, Pancreatic neuroendocrine tumors, Gastrointestinal stromal tumors
Temsirolimus	*FKBP1A* (direct), *MTOR* (indirect)	Lymphomas, Leukemia, Renal cancer
Everolimus	*FKBP1A* (direct), *MTOR* (indirect)	Breast cancer, Renal cancer, Progressive pancreatic neuroendocrine tumors

At the same time, the radiation therapy utilizing X-ray irradiation for many decades remains an established method of choice for all the above mentioned cancer types, including neuroblastoma and ovarian cancer [10, 11]. The dosage used typically varies between 40 and 60 Gy divided into daily fractions of 1.8–10 Gy depending on tumor size, metastases, invasiveness and possible side effects [12, 13]. In many clinical protocols, the radiation therapy accompanies treatment with the protein kinase inhibitor drugs, in different combinations [14–17]. Since the clinical trials of TKIs and rapalogs for the ovarian cancer and the neuroblastoma are still in progress, we aimed to investigate if this treatment may cooperate or interfere with the radiation therapy, thus altering its efficiency.

Using the two established human cancer cell lines (ovarian carcinoma SKOV-3 and neuroblastoma NGP-127), we modeled acquired resistance to three TKIs: Sorafenib, Pazopanib, Sunitinib, and two rapalogs: Everolimus and Temsirolimus. It should be mentioned, however, that the specificities of Temsirolimus and Everolimus are still under debate: although the pharmaceutical manufacturers state that they directly inhibit MTOR serine/threonine kinase complex, thus repressing AKT signaling and downregulating cell growth and survival, the detailed mechanism of their action may be more sophisticated because the drugs appear to act via an intermediate molecule - FKBP12 receptor protein, that does inhibit MTOR complex, but also has many other molecular targets, such as the TGF-beta receptor [18]. In contrast, the TKIs Sorafenib, Pazopanib and Sunitinib directly inhibit the tyrosine kinase activities of their target proteins (Table 1).

The cells were grown on gradually increasing concentrations of the five target anticancer drugs for 20 weeks. Every four weeks, we collected cell aliquots and profiled gene expression by microarrays. After 4 months of culturing, the half-inhibitory concentrations (IC_50_) increased by 25 - 186% for the particular combinations of the drugs and cell types. We next subjected cells to 10 Gy irradiation, a dose frequently used in clinical radiation therapy [19, 20]. For the ovarian cancer SKOV-3, but not neuroblastoma NGP-127 cells, for the TKIs Sorafenib, Pazopanib and Sunitinib, we noticed statistically significant increase in capacity to repair radiation-induced DNA double strand breaks compared to naïve control cells not previously treated with the TKIs. These effects were not observed for the Everolimus and Temsirolimus drugs. The potentiation on radiation therapy was linked with the increased activation of the DNA repair molecular pathway “ATM Pathway (DNA repair)” in the TKI-treated SKOV-3, but not NGP-127 cells. Our results provide a new cell culture model for studying anti-cancer therapy efficiency and evidence there may be a radioresistance emerging as a side effect of previous treatment with TKIs.

## RESULTS

### Cells with drug-resistant phenotype

We cultured the ovarian carcinoma SKOV-3 and neuroblastoma NGP-127 cells on the DMEM supplemented with 10% FBS, with the addition of TKIs and rapalogs, or, for the control experiments - without drugs added. The following concentrations were used: Sorafenib; 10 μM, Pazopanib; 25 μM, Sunitinib; 6 μM, Everolimus; 20 μM, Temsirolimus; 15 μM. Every four weeks, we took cell culture aliquots for gene expression analysis and performed MTT tests to establish the half-inhibitory concentration (IC_50_) for each cell line in order to evaluate acquired resistance (gene expression data deposited in the GEO database under accession numbers GSE97750 и GSE97751). After 20 weeks of culturing with drugs, we found that most of the cell types developed drug-resistant phenotypes for the TKIs (25-174% increase of IC_50_), and for the rapalogs (47-186% increase of IC_50_) (Table 2; Supplementary Figure 1).

**Table 2:** IC_50_ changes for the cell lines cultured on the anticancer target drugs

Incubation time	IC_50_ μM (%)				
	Sunitinib	Sorafenib	Pazopanib	Temsirolimus	Everolimus
**NGP-127 cells**					
Control	3.1	5.5	12	11.8	15.5
4 weeks	2.4 (-20%)	6.2 (14%)	13.9 (16%)	12.3 (5%)	16.4 (6%)
8 weeks	3.3 (9%)	6.8 (25%)	Not detected	14.7 (25%)	18.2 (18%)
12 weeks	4.9 (59%)	8.1 (48%)	14.7 (23%)	15.4 (31%)	19.6 (27%)
16 weeks	10.8 (250%)	7.9 (44%)	22.4 (87%)	27.9 (137%)	43 (178%)
20 weeks	8.4 (174%)	9 (64%)	24.4 (104%)	33.7 (186%)	37.9 (145%)
**SKOV-3 cells**					
Control	3	9.6	≥50	17	17.6
4 weeks	3 (0%)	10.2 (7%)	Not significant	15.8 (-7%)	19.5 (11%)
8 weeks	3.6 (20%)	10.7 (12%)	Not significant	17.5 (3%)	18.6 (6%)
12 weeks	Not detected	Not detected	Not significant	Not detected	Not detected
16 weeks	4.1 (38%)	13.2 (38%)	Not significant	15.4 (-9%)	18.6 (6%)
20 weeks	6 (100%)	12 (25%)	Not significant	24.9 (47%)	30.9 (76%)

### Culturing with target drugs affects activation of DNA repair pathways

**Figure 1:**
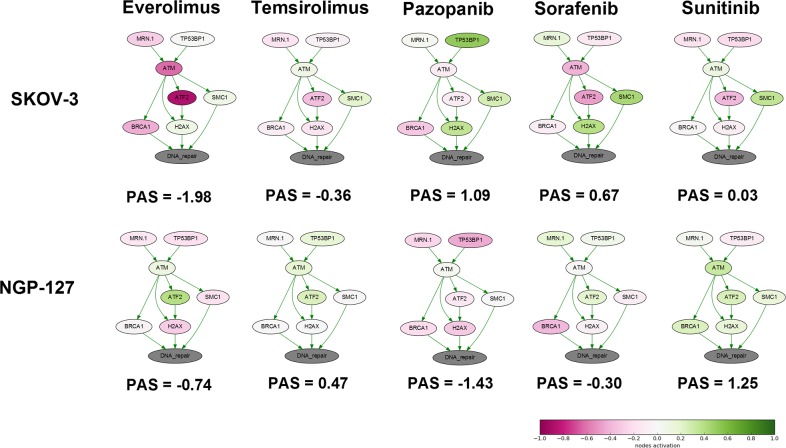
Schematic representation of alterations in “ATM Pathway (DNA repair)” molecular pathway after 4 weeks of incubation with target drugs The pathway is shown as an interacting network, where green arrows indicate activation, red arrows – inhibition. Pathway Activation Strength score is shown for each sample. Color depth corresponds to the logarithms of the case-to-normal (CNR) expression rate for each node, where “normal” is a geometric average between control samples. Exact CNR values are provided in Supplementary Table 2.

We next analyzed the changes in the activation of DNA repair pathways in relation to culturing with target anticancer drugs. To this end, we applied the OncoFinder molecular pathway analysis algorithm and database to profile the activation of 35 DNA repair pathways, namely, ATM Pathway, BRCA1 Pathway, Mismatch Repair Pathway, NHEJ mechanisms of DSBs repair Pathway and others and calculated Pathway Activation Strength (PAS) score for each pathway (supplementary Table 1) [21]. We also analyze branches of some large pathways separately to detect minor but functional pathway alterations. This method was found useful to suppress bias and batch effects associated with the high throughput profiling of gene expression [22]. This approach was published to assess the activation of signaling [23] and metabolic [24] pathways in numerous objects related to human pathology and aging [25], such as the cancer tissues [26] and cell lines [27], asthma [28], fibrosis [29] and progeria [30]. For pathway activation calculations, for each time point, we took the naïve cells gene expression data as the controls to normalize changes associated with the culturing in the presence of target anticancer drugs. It appeared that deviation of PAS values between the samples for DNA repair pathways was higher at early time-points (Supplementary Figure 2 and Supplementary Figure 3). Thus drug susceptibility observed after few month of incubation may be linked with alterations of certain pathways occurred during first weeks of treatment. We focused at 4 week time-point and found that “ATM Pathway (DNA repair)” pathway was increased in cells cultured with TKIs compared to rapalogs in SKOV-3 cell line (Figure 1). Surprisingly there was no such difference for NGP-127 cell line. We provide extended visualization for these pathways: each pathway is presented as an interacting network, where color depth of a vertex shows changes in the expression level of the corresponding gene, and edge color shows nature of the protein-protein interaction (activation or inhibition). We built such a graph for each pair “cell line – drug”. As shown on Figure 1, culturing SKOV-3 cells with TKIs increased expression level of *SMC* and *H2AX* (except Sunitinib) genes – members of the ATM Pathway (DNA repair). ATM is activated by DNA double-strand breaks and activates DNA damage checkpoints by phosphorylating Chk2, p53, SMC1 and H2AX.

### Measuring the sensitivity to X-ray irradiation

We next subjected the cells cultured with drugs for 20 weeks and the corresponding naïve cells to X-ray irradiation. The irradiation was performed at a dose of 10 Gy. This dose was chosen because it is most frequently used as a daily fraction during stereotaxic radiosurgery of solid tumors [20, 31].

Ionizing radiation (IR) results in many kinds of DNA damage [32]. Double strand DNA breaks (DSBs) constitute a relatively small fraction of these damages, but they act as the trigger for determining the cell fate [33]. Cellular response to the IR directly depends on the number of accumulated DSBs and may result in arrest of the cell cycle, upregulation of DNA repair, or programmed cell death [34]. The number of DSBs, therefore, is the prognostic biomarker of the radiation-induced response and cell survival after treatment with IR.

As the criterion of radiation sensitivity, we took residual DSBs (24 hours after IR) that represent complex non-repaired DNA lesions potentially lethal for the cell. The number of the residual foci of the DSB-binding proteins, such as the phosphorylated histone H2AX (γH2AX), is known to correlate with the cell survival after IR and is used as the marker of radioresistance in cell populations [35–38]. Recently, more accurate assays have been developed that use quantization of both H2AX foci and foci of serine 1981-phosphorylated active ATM (Ataxia Telangiectasia Mutation) kinase (pATM) [39]. This kinase may be activated activated in response to DSB and, in turn, phosphorylates histone H2AX [40].

### Quantitative analysis of γH2AX foci

**Figure 2:**
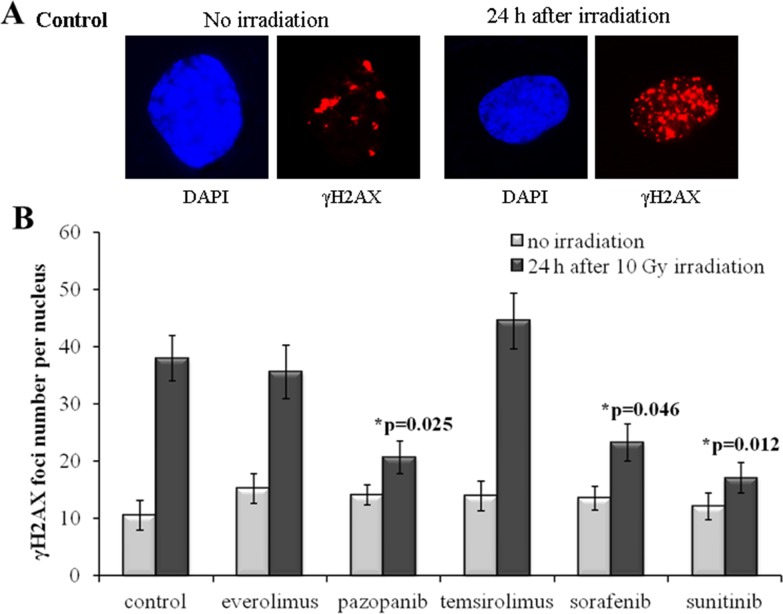
γH2AX foci in SKOV-3 cells after irradiation (10 Gy) Cells were analyzed 24 hours after irradiation. **(A)** Immunofluorescence microphotographs of SKOV-3 cells with and without irradiation stained with DAPI and monoclonal antibody against γH2AX. **(B)** Quantification of γH2AX foci in naïve SKOV-3 cells and cells adapted to target drugs for 5 month. The data are presented as average and standard error.

Our results evidence that for the SKOV-3 cells, the long-term (20 weeks) previous culturing with the tyrosine kinase inhibitors (Sorafenib, Pazopanib and Sunitinib) enhances radioresistance. Compared to the controls (naive cells), for the TKI-grown cells, the number of residual γH2AX foci after IR dropped by ∼35-50% (p=0.025; 0.046; 0.012 for Pazopanib, Sorafenib and Sunitinib, respectively). At the same time, the irradiated cells previously treated with rapalogs were not peculiar compared to the controls (Figure 2). However, for the non-irradiated cells, we detected similar proportions of spontaneous γH2AX foci in the controls and both the TKI- or rapalog-treated cells. Previous growth on TKI-containing medium was, therefore, a factor promoting the ability of SKOV-3 cells to repair their DSBs and, consequently, increasing radioresistance.

**Figure 3:**
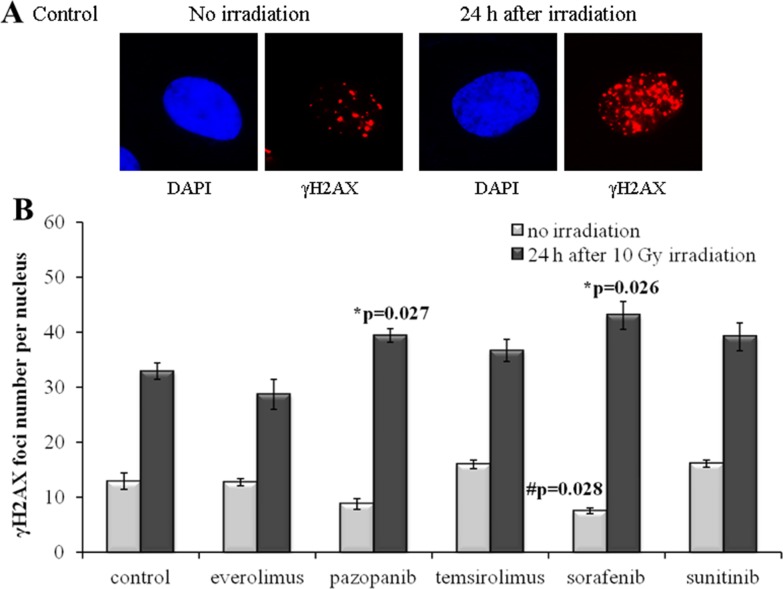
γH2AX foci in NGP-127 cells after irradiation (10 Gy) Cells were analyzed 24 hours after irradiation. **(A)** Immunofluorescence microphotographs of NGP-127 cells with and without irradiation stained with DAPI and monoclonal antibody against γH2AX. **(B)** Quantification of γH2AX foci in naïve NGP-127 cells and cells adapted to target drugs for 5 month. The data are presented as average and standard error.

In contrast to SKOV-3, culturing of the NGP-127 cells with the same drugs for 20 weeks did not induce radioresistant phenotypes (Figure 3). There was even a low (∼20%) yet statistically significant increase in the IR sensitivity for the cells grown on Pazopanib and Sorafenib TKIs (Figure 3). Interestingly, long term culturing of NGP-127 cells with Pazopanib and Sorafenib also ∼25-40% reduced numbers of spontaneous γH2AX foci in non-irradiated cells, which was statistically significant for the Sorafenib lineage (p=0.028; Figure 3).

**Figure 4:**
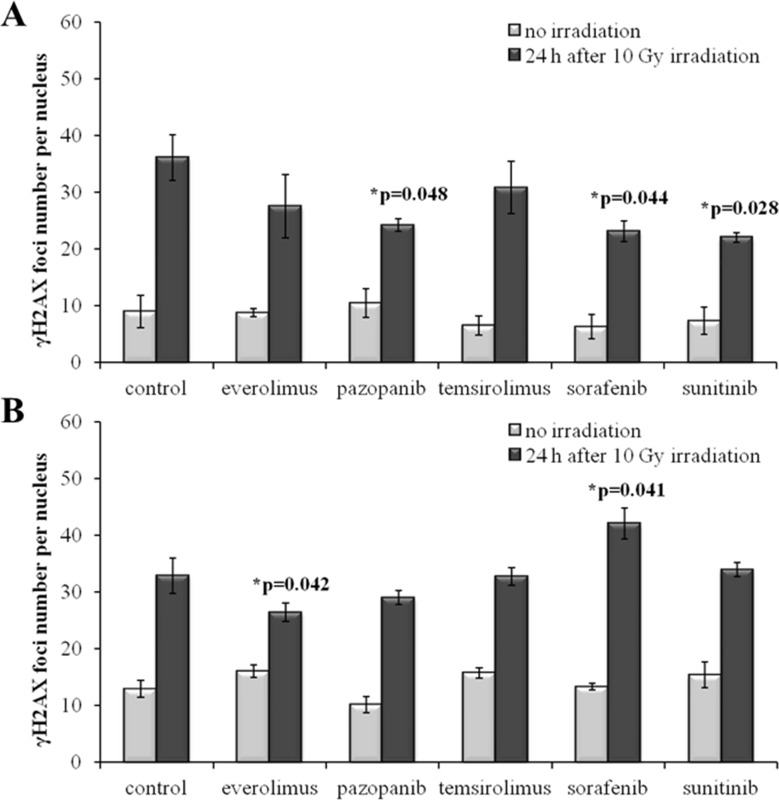
γH2AX foci in cells after irradiation (10 Gy) Resistant cells were irradiated 48 hours after removal of target drugs from the media. Cells were analyzed 24 hours after irradiation. **(A)** Quantification of γH2AX foci in naïve SKOV-3 cells and cells previously adapted to target drugs for 5 month. **(B)** Quantification of γH2AX foci in naïve NGP-127 cells and cells previously adapted to target drugs for 5 month. The data are presented as average and standard error.

The radiation therapy of cancer is typically applied after cancellation of the drug treatment for a patient as the combination of both may provoke too strong side effects. To monitor the IR effects in a more realistic model, we irradiated the drug-treated SKOV-3 and NGP-127 cells 48 hours after removal of the target drugs from the media. Twenty-four hours after 10 Gy irradiation, the cells were monitored and we still could identify statistically significant ∼35% decrease of induced γH2AX foci for the SKOV-3 cells previously treated with the TKIs, but not with the rapalogs (Figure 4A). As before, this trend was not effective in the NGP-127 cells, which showed increased numbers of foci for the Sorafenib lineage (p=0.041; Figure 4B).

### Quantitative analysis of pATM foci

**Figure 5:**
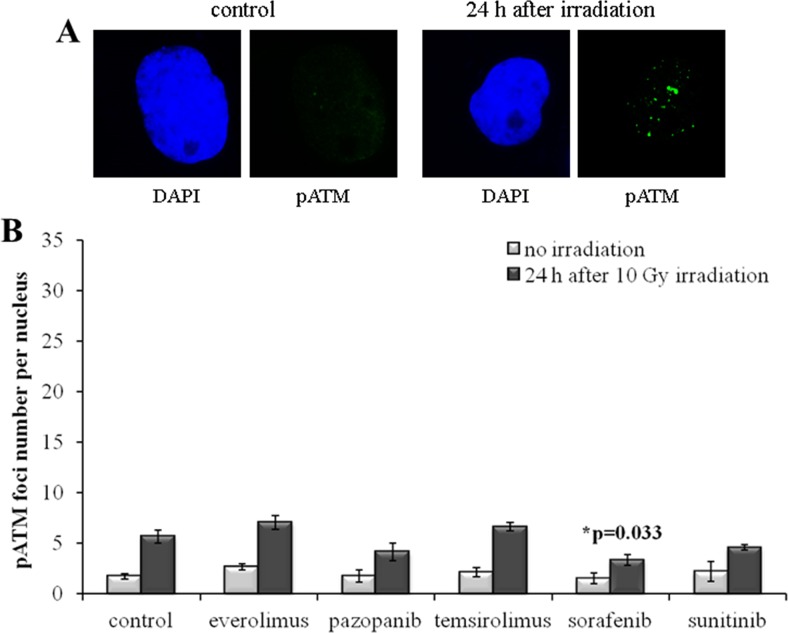
pATM foci in SKOV-3 cells after irradiation (10 Gy) Cells were analyzed 24 hours after irradiation. **(A)** Immunofluorescence microphotographs of SKOV-3 cells with and without irradiation stained with DAPI and monoclonal antibody against phosphorylated ATM protein. **(B)** Quantification of pATM foci in naïve SKOV-3 cells and cells adapted to target drugs for 5 month. The data are presented as average and standard error.

**Figure 6:**
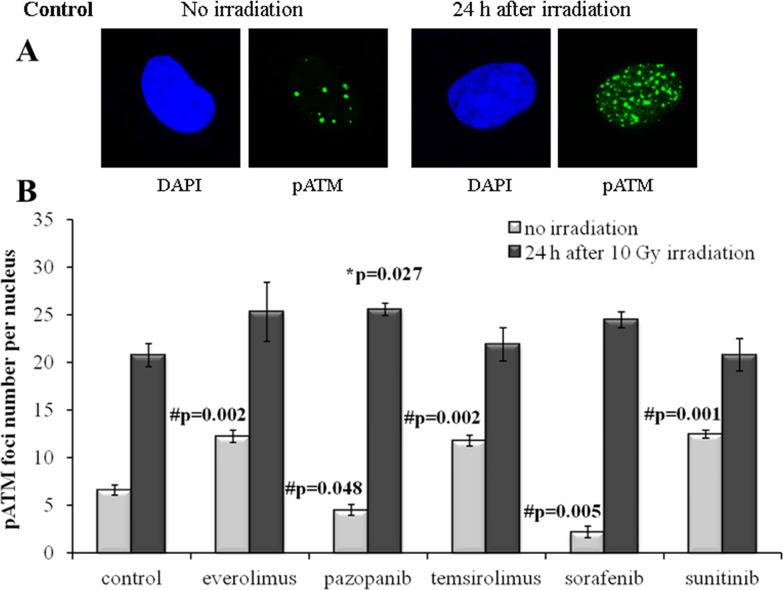
pATM foci in NGP-127 cells after irradiation (10 Gy) Cells were analyzed 24 hours after irradiation. **(A)** Immunofluorescence microphotographs of NGP-127 cells with and without irradiation stained with DAPI and monoclonal antibody against phosphorylated ATM protein. **(B)** Quantification of pATM foci in naïve NGP-127 cells and cells adapted to target drugs for 5 month. The data are presented as average and standard error.

In order to independently validate the results obtained with quantization of the γH2AX foci, we next assayed the cells for the DSB-activated form of ATM kinase (pATM). We found that the SKOV-3 cells have much lower rate of both spontaneous and radiation-induced pATM foci compared to the NGP-127 cells (Figures 5, 6). Similarly to the γH2AX results, the irradiated SKOV-3 cells previously cultured on the TKIs, but not on the rapalogs, showed ∼25-50% decrease of the relative number of IR-induced pATM foci, which suggested increased radioresistance. This tendency was seen for all the TKIs tested, being statistically significant only for the Sorafenib lineage (Figure 5).

For the NGP-127 cells, we detected high rate of spontaneous pATM foci (6.6±0.5 foci per nucleus). Long term culturing of these cells with the target drugs resulted in the opposite effects on the pATM foci accumulation. The pre-treatment with Pazopanib and Sorafenib decreased the number of spontaneous pATM foci, whereas culturing with Sunitinib unexpectedly was shown to increase their number (Figure 6). Both rapalogs (Everolimus and Temsirolimus) could also increase the appearance of spontaneous pATM foci in these cells (Figure 6). However, for the IR-induced pATM foci we found ∼20% increase in the appearance for the Pazopanib lineage (p=0.027).

**Figure 7:**
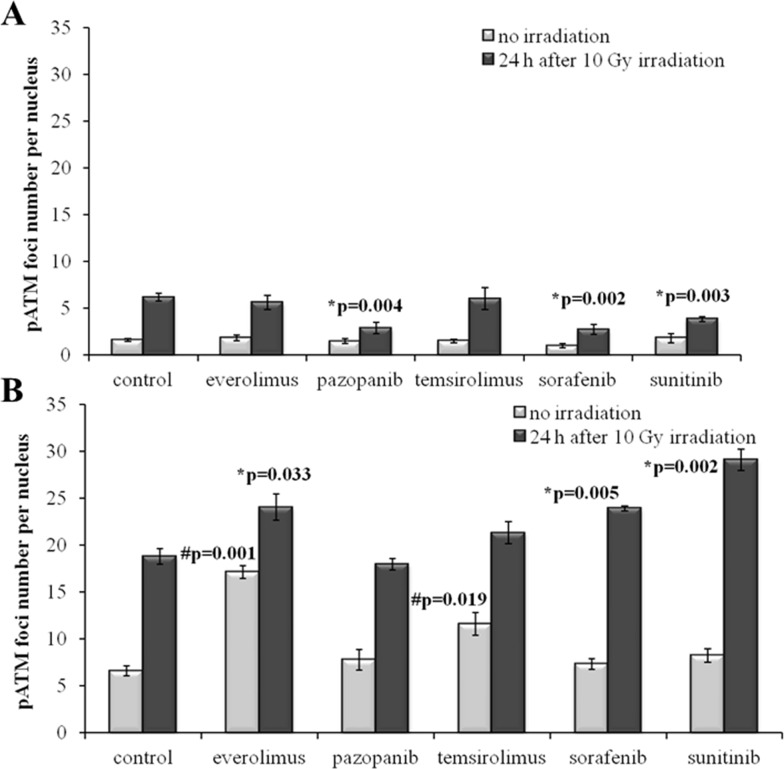
pATM foci in cells after irradiation (10 Gy) Resistant cells were irradiated 48 hours after removal of target drugs from the media. Cells were analyzed 24 hours after irradiation. **(A)** Quantification of pATM foci in naïve SKOV-3 cells and cells previously adapted to target drugs for 5 month. **(B)** Quantification of pATM foci in naïve NGP-127 cells and cells previously adapted to target drugs for 5 month. The data are presented as average and standard error.

We next measured IR-induced pATM foci in the cells 48 hours after removal of drugs. Again, for all the SKOV-3 lineages previously cultured with the TKIs, but not with the rapalogs, we detected ∼2-fold statistically significant decrease of the proportion of IR-induced foci (p=0.004; 0.002; 0.003 for Pazopanib, Sorafenib and Sunitinib, respectively). This data strongly supports our previous findings on the increase of radioresistance in these cells linked with the growth in presence of the TKIs (Figure 7A).

In contrast, the NGP-127 lineages grown on the anticancer drugs showed only increased sensitivity to the IR, which was statistically significant for the Everolimus, Sorafenib and Sunitinib (Figure 7B). We found also increased numbers of spontaneous pATM foci in the NGP-127, which were previously incubated with the rapalogs (Figure 7B).

Taken together, our data indicate that there is a clear-cut influence of the anticancer target drugs on the radiation resistance, which is cell type-specific. In the SKOV-3 cells, the TKIs, but not rapalogs, could strongly potentiate the resistance to IR, whereas in the NGP-127 cells both rapalogs and TKIs showed the ability to slightly decrease it.

## DISCUSSION

These findings evidence that the long-term culturing with the TKIs can result in a positive selection of radioresistant cells, or that it may cause molecular changes leading to growth of IR resistance in the SKOV-3 cells. In contrast, treatment with rapalogs did not result in such resistance. This is in accordance with recent findings of Chen et al., who showed that Rapamycin suppresses homologous recombination and nonhomologous end joining, two major DNA DSB repair pathways [41]. Moreover, inhibition of DSB repair system with PI3K/mTOR inhibitor NVP-BEZ235 attenuated the repair of ionizing radiation-induced DNA damage in glioblastoma model [42]. However, mechanisms of developing radiation resistance seem to be different across cell lines. For example, apoptotic response to radiation in SKOV-3 cells was lower than in HeLa cells, when *CHK1* or *DNA-PK* genes were interfered in both cell lines with short hairpin RNA [43]. Our findings are also in line with earlier work by Li and co-authors, who showed that even short term pre-culturing of hepatocellular carcinoma cell lines with Sorafenib resulted in significantly fewer cells with γ-H2AX foci after subsequent irradiation [44].

Enhanced selection of radioresistant cells treated with TKIs, when compared to rapalogs, may be linked with increased expression of *SMC* or *H2AX* genes. Indeed, the SMC proteins promote DNA repair and genomic stability [45]. In addition, it was shown recently that Sunitinib induces cell-cycle arrest and DNA repair in breast cancer cells [46]. This may be another mechanism of acquiring radioresistance by TKI-treated SKOV-3 cells.

When studying global pathway activation changes associated with the drug resistance, we found that they had much greater amplitude in the SKOV-3 than in the NGP-127 cells (Supplementary Figure 2 and Supplementary Figure 3). The highest variance was seen for the 4^th^ week of culturing with drugs, and was largely decreasing with time up to the end of the experiment. This evidences that the most stressful period of adding new cytotoxic agents to the cells is linked with the most extensive reorganization of intracellular molecular signaling. This may suggest establishing of a novel equilibrium in gene regulatory networks during adaptation to the anticancer drugs that is more similar to the initial state at the end of the culturing rather at the beginning. Our data also demonstrate that the complex adaptation of the cells to the target drugs may have contradictory yet strong and statistically significant effects on the efficiency of DNA repair, as evidenced by the different double strand DNA break repair efficiencies after IR marked by the γH2AX and pATM foci. Since there is a clear cell type-specific link between the efficiency of IR and previous chemotherapy, our data accentuate the importance of organizing thorough clinical studies for each specific cancer type before generally accepting the specific schemes of the sequential target- and radiation therapies, especially including the TKIs treatment.

## MATERIALS AND METHODS

### Biosamples

In this study, we used two human cell lines to profile gene expression and responses to anticancer drugs. The NGP-127 and SKOV-3 cells were cultured on Dulbecco's modified Eagle's medium (DMEM; Gibco, USA) supplemented with 10% heat-inactivated fetal bovine serum (HyClone, USA), 100 mkg/ml penicillin (Sigma, USA), 100 U/ml streptomycin (Sigma, USA) and 2mM *L*-glutamine (Sigma, USA) at 37°C and 5% CO_2_. The cells were grown in 25 cm^2^ or 75 cm^2^ flask (Greiner, Germany) and passaged for every 72 hours.

### Cell culturing with TKIs and viability assay

NGP-127 and SKOV-3 cells resistant to five target anticancer drugs were generated by growing naive cells serially treated with an increasing doses of Sunitinib up to 6 μM, Pazopanib up to 25 μM, Sorafenib up to 10 μM, Everolimus up to 20 μM and Temsirolimus up to 15 μM (Selleckchem, USA). After continuous culture in complete medium supplemented with the appropriate concentrations of the drugs for >10 weeks, these cells were used as resistant cell lines for all subsequent experiments.

We evaluated cell viability by using MTT (3-[4,5-dimethylthiazol-2-yl]-2,5-diphenyltetrazolium bromide) test [47]. Adherent cells were trypsinized, and washed twice with DMEM by 5-min centrifugation at 300*g*. Aliquots of cells were counted in hemocytometer. Cells were seeded in 96-well plates (Greiner, Germany) in growth medium at 2.000-4.000 cells per well, depending on the cell line used. The plates were pre-incubated for 18 h before the addition of testing components. The following drugs were tested (purchased at Selleckchem, USA): Pazopanib, Sunitinib, Sorafenib, Everolimus and Temsirolimus. For every cell line, the drugs were tested in the following concentrations: 0, 0.8, 1.56, 3.1, 6.25, 12.5, 25 and 50 μM. All the experiments were made in quadruplicate. After addition of the testing components, the plates were incubated for 72 h and then plates were centrifuged at 300 g for 10 min, followed by the removal of supernatant. 30 μl of 0.5 mg/ml solution of MTT (Sigma, USA) was added to each well, and the plates were incubated for 2–4 h, then 100 μl of DMSO was added to each well for formazan crystals dissolving. The optical densities (OD) at 540 nm were measured using a plate reader Multiscan FC (ThermoScientific, USA). Cell viability was calculated using the formulae: (OD treated cells – OD blank)/(OD control cells – OD blank) × 100%, where OD blank means OD in control wells containing no cells. IC_50_ values were deduced from Dose–response curves using SigmaPlot software (Systat Software Inc., USA). Dose-response curves and IC_50_ values are given in Table 2 and Supplementary Figure 1.

### Synthesis of microarrays

B3 microarray synthesizer (CustomArray, USA) was used for forty nucleotides-long oligonucleotide probe synthesis on CustomArray ECD 4×2K/12K slides. Synthesis was performed according to the manufacturer's recommendations. Two replicates of total 6020 unique oligonucleotide probes specific to 3706 human gene transcripts were placed on each chip. Chip design was performed using Layout Designer software (CustomArray, USA). For the custom microchip, we used original oligonucleotide probe sequences of the Illumina HT 12 v4 platform.

### Library preparation and hybridization

Complete Whole Transcriptome Amplification WTA2 Kit (Sigma) was used for reverse transcription and library amplification. Manufacturers protocol was modified by adding to amplification reaction dNTP mix containing biotinylated dUTP, resulting to final proportion dTTP/biotin-dUTP as 5/1. Microarray hybridization was performed according to the CustomArray ElectraSense™ Hybridization and Detection protocol. Hybridization mix contained 2.5 ug of labeleled DNA library, 6X SSPE, 0.05% Tween-20, 20mM EDTA, 5x Denhardt solution, 100 ng/ul sonicated calf thymus gDNA, 0,05% SDS. Hybridization mix was incubated with chip overnight at 50°C. Hybridization efficiency was detected electrochemically using CustomArray ElectraSense™ Detection Kit and ElectraSense™ 4×2K/12K Reader.

### Initial processing of microarray data

Probe signals were geometrically averaged, thus obtaining expression value for each specific type of the probe. Then quantile normalization [48] was performed using the ‘preprocessCore’ R package [49]. Gene expression data were deposited in Gene Expression Omnibus database with the accession numbers GSE97750 and GSE97751.

### Functional annotation of gene expression

The Oncofinder knowledge base was used to determine structures of intracellular molecular pathways linked with DNA repair, as described previously [50]. We applied the original OncoFinder algorithm [21] for functional annotation of the primary expression data and for calculating pathway activation strength (PAS) scores and cancer-to-normal ratios (CNRs). CNRn is the ratio of the expression levels of a gene n in the sample under investigation to the average expression in the control group of samples. In this study, the PAS scores were obtained according to [21]. PAS can take both positive and negative values meaning over- or underactivation relative to control tissue. Results for the 35 molecular pathways obtained for each sample are shown on Supplementary dataset 1.

### Irradiation

Cells were exposed to 200 kV X-rays at a dose rate of 0.85 Gy/min (2.5 mA, 1.5 mm Al filter) using RUB RUST-M1 X-irradiator (Russia). Throughout the irradiation, cells were maintained at 4°C using a thermo-granules Lab Armour (Life Technologies, USA). The error of exposure dose were calculated to be within 10%. Cells were returned to normal growth conditions immediately after irradiation and maintained for various periods of time before fixation.

### Immunofluorescence microscopy

Cells were fixed on coverslips in 4% paraformaldehyde in PBS (pH 7.4) for 20 min at room temperature followed by two rinses in PBS and permeabilization in 0.3% Triton-X100 (in PBS, pH 7.4) supplemented with 2% bovine serum albumin (BSA) to block non-specific antibody binding. Cells were then incubated for 1 hour at room temperature with primary rabbit monoclonal antibody against γH2AX (clone EP854(2)Y, Merck-Millipore, USA) and primary mouse monoclonal antibody against phosphorylated ATM protein (clone 10H11.E12, Merck-Millipore, USA) which were diluted in PBS (1:200 and 1:400, respectively) with 1% BSA. Following several rinses with PBS, cells were incubated for 1 hour at room temperature with secondary antibodies IgG (H+L) goat anti-mouse (Alexa Fluor 488 conjugated, dilution 1:600; Merck-Millipore, USA) and goat anti-rabbit (rhodamine conjugated, dilution 1:400; Merck-Millipore, USA) diluted in PBS (pH 7.4) with 1% BSA. Coverslips were then rinsed several times with PBS and mounted on microscope slides with ProLong Gold medium (Life Technologies, USA) with DAPI for DNA counter-staining. Cells were viewed and imaged using Nikon Eclipse Ni-U microscope (Nikon, Japan) equipped with a high definition camera ProgRes MFcool (Jenoptik AG, Germany). Filter sets used were UV-2E/C (340-380 nm excitation and 435-485 nm emission) and Y-2E/C (540-580 nm excitation and 600-660 nm emission). At least 200 cells per data point were imaged.

### Statistical analysis

Statistical analyses of the immunocytochemical data were conducted using the Statistica 8.0 software (StatSoft). The results are presented as means of three independent experiments ± standard error. Statistical significance of irradiation analysis was tested using the Student t-test. Statistical significance of gene expression changes was assessed with two-sigma rule: the gene was considered differentially expressed if its measured transcription level was outside the interval of mean expression ± two standard deviations of expression in control samples. We did five independent biological replicates to measure gene expression in control samples.

## SUPPLEMENTARY FIGURES AND TABLES





## Abbreviations

PAS: Pathway Activation Strength
TKIs: tyrosine kinase inhibitors
TKI: tyrosine kinase inhibitor
MTT: 3-[4,5-dimethylthiazol-2-yl]-2,5-diphenyltetrazolium bromide
CNR: Case-to-Normal Ratio
IR: Ionizing radiation
DSBs: Double strand DNA breaks
IC_50_: The half maximal inhibitory concentration
Gy: Gray
