# Supplementary material for: Acquired resistance to tyrosine kinase inhibitors may be linked with the decreased sensitivity to X-ray irradiation
Source: Oncotarget. 2017 Dec 27;9(4):5111–24. doi: 10.18632/oncotarget.23700 (PMC5797037; doi:10.18632/oncotarget.23700)
Supplement: Supplementary file 1 [file oncotarget-09-5111-s001.pdf]

Acquired resistance to tyrosine kinase inhibitors may be linked with the decreased sensitivity to X-ray irradiation

SUPPLEMENTARY MATERIALS

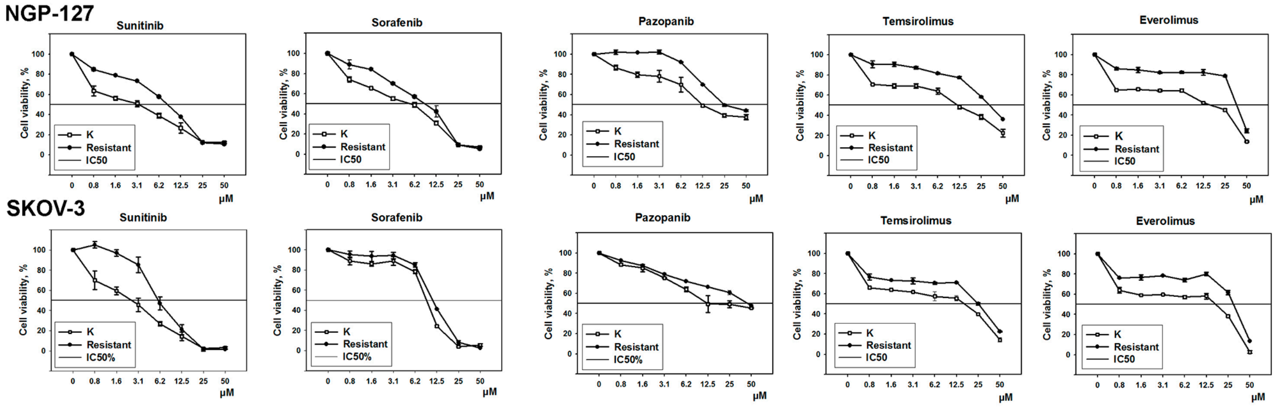

Supplementary Figure 1: Viability of SKOV3 and NGP-127 cells treated with different concentrations of target drugs. Viability and IC_{50} were measured with MTT test. Each graph shows dose-response curves for naïve (K) and resistant cells, which were treated with corresponding drug for 5 month.

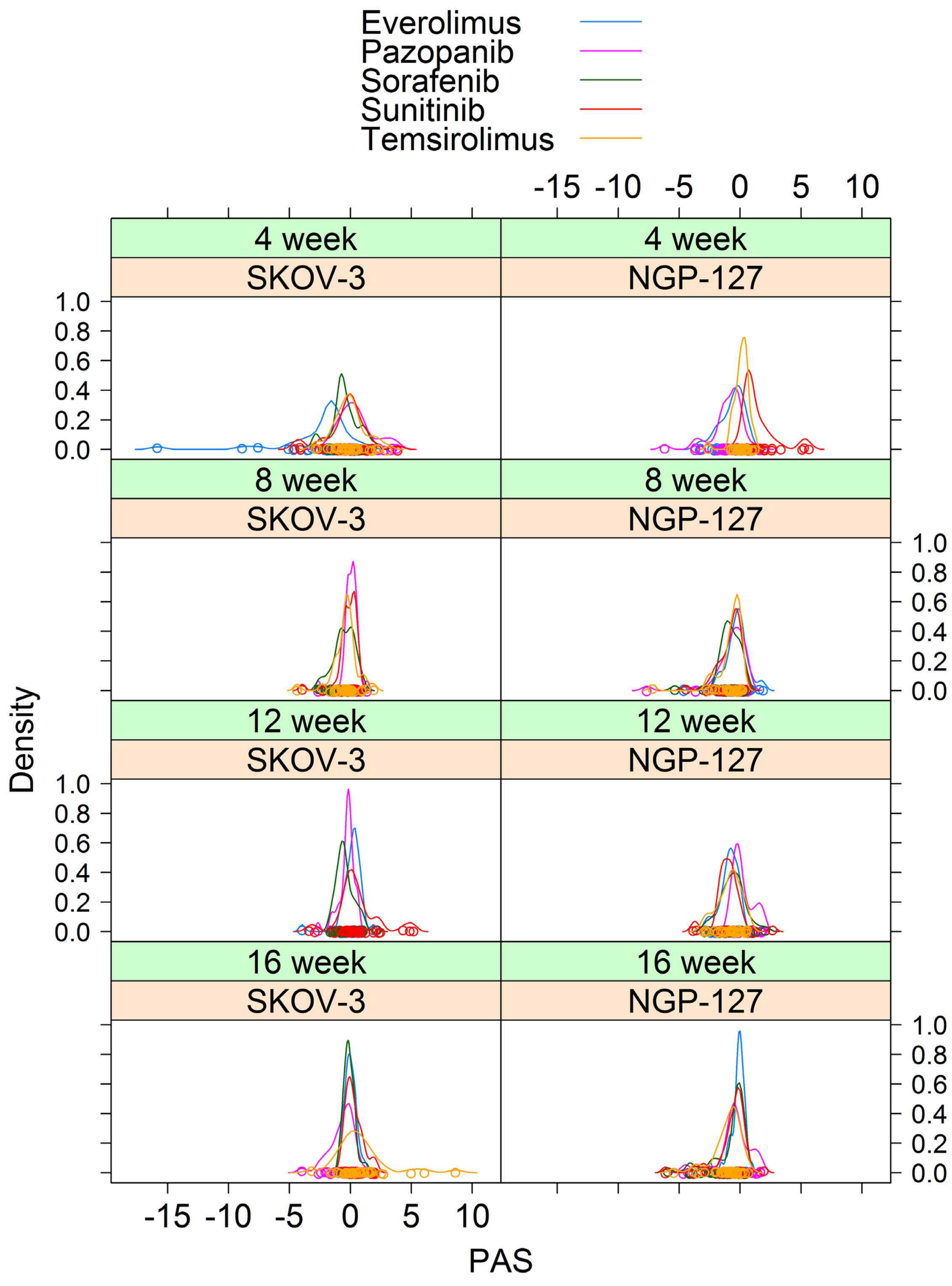

Supplementary Figure 2: Distribution of PAS values over time. A density plot was built for each cell line for each drug and for each time-point. Each curve shows density of Pathway Activation Scores for 35 DNA repair pathways. Density plots were built with Lattice R package.

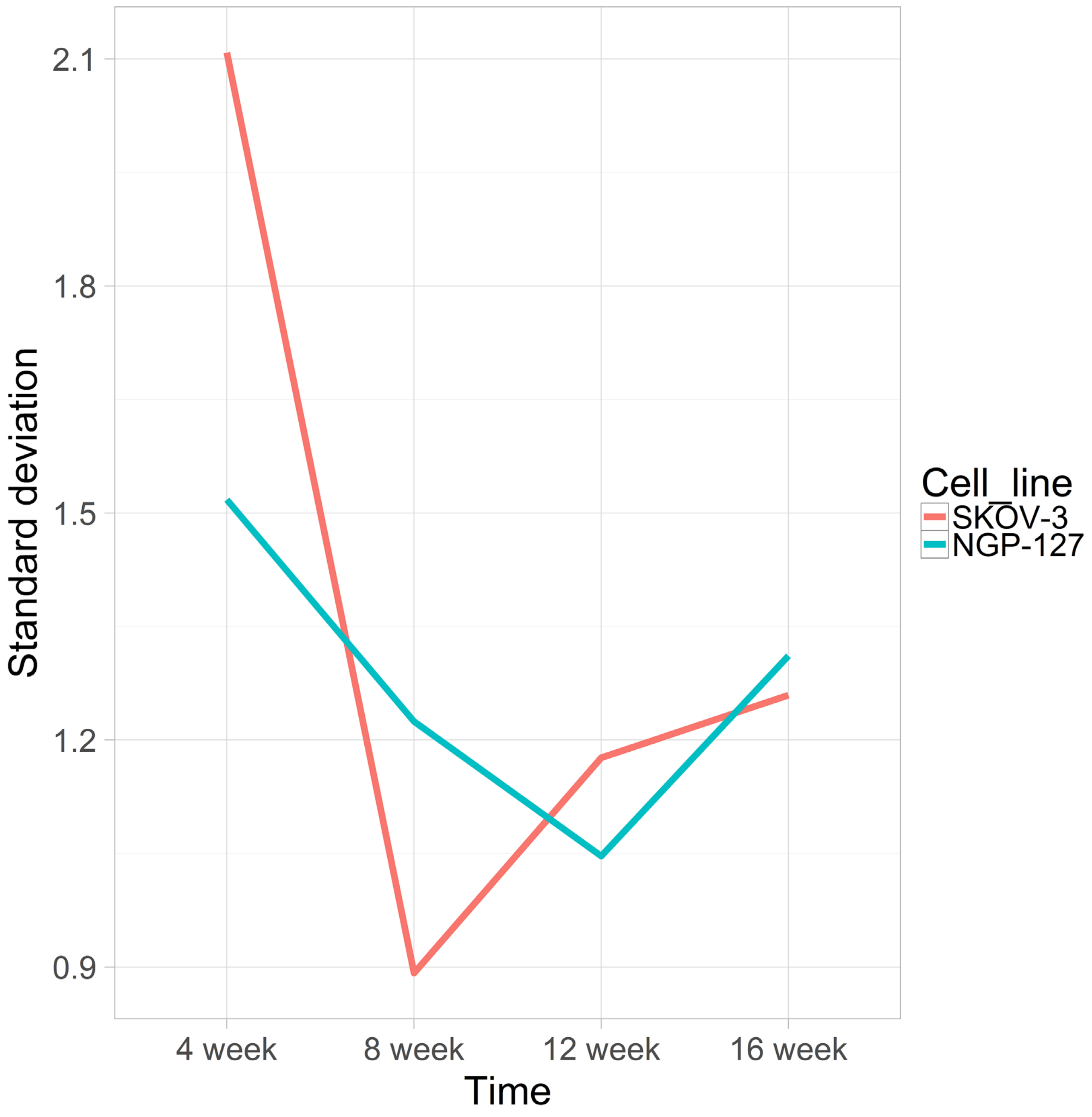

Supplementary Figure 3: Standard deviation of PAS values over time. PAS for different drugs were pooled to estimate standard deviation at each time point, thus every point represents standard deviation of 175 Pathway Activation Scores.

Supplementary Table 1: Pathway Activation Scores of 35 DNA repair pathways after 4 weeks of incubation with target drugs.

See Supplementary File 1

Supplementary Table 2: Exact CNR values of genes, which are involved in the "ATM_Pathway_DNA_repair" pathway shown on Figure 1

	Gene	Everolimus	Temsirolimus	Pazopanib	Sorafenib	Sunitinib
	ATF2	0,41 (1)	0,69 (1)	0,89 (0)	0,62 (1)	0,67 (1)
	ATM	0,53 (1)	1,08 (0)	0,89 (0)	0,66 (1)	1,11 (0)
	BRCA1	0,65 (1)	0,9 (0)	0,73 (1)	0,91 (0)	0,97 (0)
	H2AFX	***1,09 (0)***	***0,85 (0)***	***1,42 (1)***	***1,48 (1)***	***0,92 (0)***
**SKOV-3**	MRE11A	0,75 (1)	0,85 (0)	1,03 (0)	1,16 (0)	0,84 (0)
	SMC1A	***0,9 (0)***	***1,78 (1)***	***1,7 (1)***	***2,07 (1)***	***2,27 (1)***
	SMC2	***1,52 (1)***	***1,11 (0)***	***1,23 (0)***	***1,45 (1)***	***1,2 (0)***
	SMC3	***0,82 (0)***	***0,74 (1)***	***0,98 (0)***	***1,14 (0)***	***0,82 (0)***
	TP53BP1	1 (0)	0,93 (0)	1,68 (1)	0,88 (0)	0,8 (0)
	ATF2	1,51 (0)	1,29 (0)	0,87 (0)	1,23 (0)	1,3 (0)
	ATM	1,12 (0)	1,2 (0)	1,03 (0)	1,01 (0)	1,37 (0)
	BRCA1	0,97 (0)	0,98 (0)	0,8 (0)	0,68 (0)	1,27 (0)
	H2AFX	***0,75 (0)***	***1 (0)***	***0,73 (0)***	***0,94 (0)***	***1,16 (0)***
**NGP-127**	MRE11A	0,86 (0)	1 (0)	0,78 (0)	1,19 (0)	1,04 (0)
	SMC1A	***0,83 (0)***	***1,05 (0)***	***1,26 (1)***	***1,11 (0)***	***0,86 (0)***
	SMC2	***0,74 (0)***	***0,93 (0)***	***0,95 (0)***	***0,79 (0)***	***1,41 (0)***
	SMC3	***0,91 (0)***	***0,93 (0)***	***0,76 (0)***	***0,85 (0)***	***1,19 (0)***
	TP53BP1	0,81 (1)	1,17 (1)	0,64 (1)	1,05 (0)	0,89 (1)

The value in brackets indicates significance of CNR. If the gene was significantly differentially expressed (see Materials and methods section) the value is 1, 0 – otherwise.
